# Applying a Health Network approach to translate evidence-informed policy into practice: A review and case study on musculoskeletal health

**DOI:** 10.1186/1472-6963-12-394

**Published:** 2012-11-14

**Authors:** Andrew M Briggs, Peter Bragge, Helen Slater, Madelynn Chan, Simon CB Towler

**Affiliations:** 1 Department of Health, Government of Western Australia, GPO Box 8172, Perth Business Centre, WA, 6849, Australia; 2 Curtin Health Innovation Research Institute, Curtin University, Perth, Australia; 3 National Trauma Research Institute, The Alfred Hospital & Monash University, Melbourne, Australia; 4 School of Physiotherapy, Curtin University, Perth, Australia; 5 Department of Rheumatology, Royal Perth Hospital, Perth, Australia

**Keywords:** Policy, Network, Community of practice, Musculoskeletal, Translation, Evidence

## Abstract

**Background:**

While translation of evidence into health policy and practice is recognised as critical to optimising health system performance and health-related outcomes for consumers, mechanisms to effectively achieve these goals are neither well understood, nor widely communicated. Health Networks represent a framework which offers a possible solution to this dilemma, particularly in light of emerging evidence regarding the importance of establishing relationships between stakeholders and identifying clinical leaders to drive evidence integration and translation into policy. This is particularly important for service delivery related to chronic diseases. In Western Australia (WA), disease and population-specific Health Networks are comprised of cross-discipline stakeholders who work collaboratively to develop evidence-informed policies and drive their implementation. Since establishment of the Health Networks in WA, over 50 evidence-informed Models of Care (MoCs) have been produced across 18 condition or population-focused Networks. The aim of this paper is to provide an overview of the Health Network framework in facilitating the translation of evidence into policy and practice with a particular focus on musculoskeletal health.

**Case presentation:**

A review of activities of the WA Musculoskeletal Health Network was undertaken, focussing on outcomes and the processes used to achieve them in the context of: development of policy, procurement of funding, stakeholder engagement, publications, and projects undertaken by the Network which aligned to implementation of MoCs.

The Musculoskeletal Health Network has developed four MoCs which reflect Australian National Health Priority Areas. Establishment of community-based services for consumers with musculoskeletal health conditions is a key recommendation from these MoCs. Through mapping barriers and enablers to policy implementation, working groups, led by local clinical leaders and supported by the broader Network and government officers, have undertaken a range of integrated projects, such as the establishment of a community-based, multidisciplinary rheumatology service. The success of these projects has been contingent on developing relationships between key stakeholders across the health system.

**Conclusions:**

In WA, Networks have provided a sustainable mechanism to meaningfully engage consumers, carers, clinicians and other stakeholders; provided a forum to exchange ideas, information and evidence; and collaboratively plan and deliver evidence-based and contextually-appropriate health system improvements for consumers.

## Background

### The need for effective alignment of evidence, policy and practice in contemporary healthcare

Given the current international and national data on chronic disease prevalence, future projections, and the associated health sequelae and healthcare costs [1-3], which clearly demonstrate an exponential escalation that is economically unsustainable, optimisation of healthcare delivery in this area is a health policy and whole-of-government imperative [4-6]. Three specific challenges are faced by policymakers in trying to optimise healthcare delivery, including (i) identifying effective evidence-based interventions to address health problems; (ii) identifying how to best integrate these into health systems; and (iii) identifying how to best drive these changes across health systems [7]. Although there is a significant body of high-level evidence demonstrating ‘what works’ in clinical practice, for example through clinical trials, clinical practice guidelines, and Cochrane systematic reviews, far less evidence is available to guide how to successfully translate such evidence into policy and widespread practice [7]. While the uptake of evidence to inform policy in some circumstances may be rapid; for example, the removal of government subsidies for vertebroplasty procedures for osteoporotic vertebral fractures in Australia in response to two recent randomised controlled trials [8,9] and a meta-analysis [10] which demonstrated no clinical benefit for the procedure; this policy response is reactive and less preferable than a standardised, incremental approach to evidence translation into policy. Notwithstanding the knowledge gap in effectively translating evidence into policy, the literature on the effectiveness of various strategies for achieving evidence integration and translation is growing. The Cochrane Effective Practice and Organisation of Care (EPOC) Group has completed 92 systematic reviews of interventions designed to improve the delivery, practice, and organisation of health care services, and protocols for a further 49 systematic reviews in this field [11]. Further, the Health Systems Evidence database contains over 4000 systematic reviews, evidence briefs and other syntheses of research evidence on governance, financial and delivery arrangements within health systems, and interventions designed to support change in health systems [12].

In a review of literature on evidence integration and translation in health care policy, Mitton et al. [13] identified a number of key strategies for achieving evidence integration, including i) face-to-face exchange between decision-makers and researchers (for example to gather information on current practices, develop relationships and broker involvement in change implementation); ii) the use of Networks and/or Communities of Practice; iii) capacity-building within health services and health delivery organisations (for example, enhancing capacity to implement health promotion programs, providing training in critical appraisal of research literature); and iv) use of steering committees to harness strategic input from local experts into future research and evidence-informed policy development. Notably, because the focus of these strategies is on the establishment of bilateral communication and stakeholder involvement, the development of trustworthy relationships between research partners was identified as a central factor to their success. The same factor was also identified by more recent primary research [14,15] and consensus opinion [16], and mirrors the benefits of involving stakeholders in policy development [17]. Haynes et al. [15] recently reported data derived from policymakers on the barriers and facilitators to effective engagement with researchers. Identifying trustworthy researchers who understood the political environment and government processes was a key challenge discussed. Given this pivotal role of relationship building, how can meaningful and effective relationships be forged between stakeholders, be fostered, and be used to drive translation of evidence into policy and practice?

### Using a Health Network approach to bridge the evidence-policy-practice gaps

One strategy to help build and strengthen critical relationships between stakeholders in healthcare is to target the disconnect between evidence and health policy and practice through the use of a Health Network or Community of Practice (CoP) approach [18,19]. A network is a partnership, collaboration or alliance between individuals and/or organisations that are connected in some way, such as through shared interests, professional roles, or organisational linkages [20,21]. A CoP uses the Network model to develop a collective, or ‘community’, to share information, solve problems and drive innovation in an area of common interest and understanding [22]. Given the greater accessibility to health services now provided by information and communication technologies such as telehealth, *e*Health, and *e*doctor, the concept of a CoP has expanded beyond the traditional face-to-face forum to include virtual CoPs, potentially reducing some of the geographic barriers and competing work and lifestyle commitments which limit opportunities for face-to-face encounters for consumers and healthcare professionals [23,24]. The formation of virtual CoPs may also open opportunities for exchange in developing nations and between jurisdictions [25].

In complex systems such as health, and especially in the context of chronic disease where system reforms are inherently multifaceted, Networks offer a potential solution to informing and implementing organisational change, and facilitating the alignment of evidence-informed policy and practice. This is achieved principally through the engagement of multidisciplinary stakeholders to form working relationships in order to identify and develop solutions to system barriers. While the concept of a Network model is not new, its application to influencing organisational change in the health sector in an integrated and formal manner is an emerging field of interest for researchers and policymakers [18]. Evidence to substantiate the effectiveness of Networks in tackling clinical health issues is accumulating [20,26-32], and a similar Network approach has been adopted in other fields, for example environmental science [33]. While discrete Networks such as clinical Networks, policy Networks, and service management Networks may evolve naturally, these Networks are potentially siloed due to disparate perceived stakeholder interests and varying scopes of influence or professional training focus. Siloed Networks may be less effective in bridging evidence-policy-practice gaps across the broader health continuum because their goals, activities and functions are perceived to be independent of one another. In contrast, the sphere of influence, and therefore effectiveness, of a multidisciplinary (or multiple-stakeholder) Network may be substantially greater [18,30,32,34] because collective efforts and diverse skill sets can be harnessed towards a common goal. The aim of this paper is to provide an overview of Health Networks in Western Australia (WA) and to describe a case study of the application of a multidisciplinary Health Network model to the development of evidence-informed policy and service delivery for consumers with musculoskeletal health conditions.

### Health Networks in Western Australia

#### Development and establishment Health Networks in Western Australia

In 2004, a Network model was established in WA ( http://www.healthnetworks.health.wa.gov.au) as a health reform strategy for the state [35]. Initially, the focus of the WA reform initiative was to establish *Clinical Networks*; engaging the clinical sector to tackle complex health issues through the development of health policy [36]. These Clinical Networks were later transformed into *Health Networks*, in recognition of the critical roles of non-clinical stakeholders such as consumers, carers, non-government organisations in the planning, delivery and evaluation of health services for Western Australians across the continuum of care. This exemplifies a shift from a siloed to a multidisciplinary Network model and underlines the fundamental premise of the *Health Network* model: that multidisciplinary partnerships are the most effective way to address complex problems and reach common goals of relevance across the health continuum [37]. The aim of the WA Health Networks is to involve all stakeholders (i.e. establish a ‘community’) with a shared interest in health (consumers, carers, clinicians, policy makers, non-government organisations, researchers, educators and so on) to interact and exchange information with a view to collaboratively plan and facilitate implementation of consumer-centred health services and policies (locally, coined *‘connect, share, improve’*); therefore, this model may also be considered a CoP approach [18,22]. Such collaboration promotes a consumer-centred focus and functions as a platform for reform through development of evidence-informed policies and programs that are context-specific, a critical aspect of successful implementation [38]; in this case, relevant to the WA health system. A key output of this collaborative process is a Model of Care; a state-wide, evidence-informed policy that clearly articulates a framework for consumer-centred health service delivery (the *right care*, at the *right time*, by the *right team*, in the *right place*) [39]. Importantly, a Model of Care contains key facilitators identified by Mitton et al. [13] in driving evidence into policy and practice. Since establishment of the Health Networks in WA, over 50 evidence-informed Models of Care have been produced across 18 condition or population-focused Networks.

#### Translation of policy into practice: a phased implementation approach

**Figure 1:**
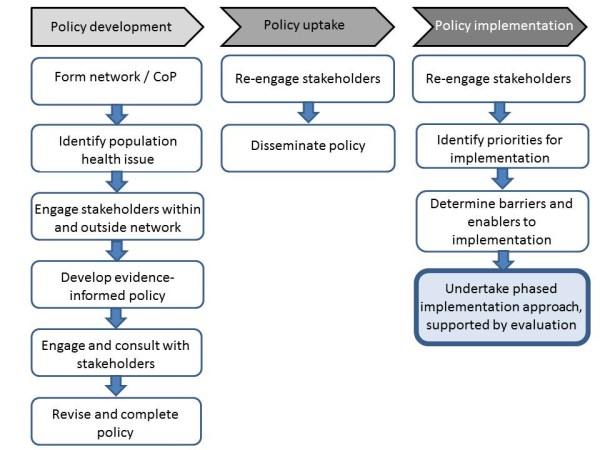
Schematic of the phases involved in Health Network policy development and implementation cycle.

Broadly, three phases are involved in translating policy into practice under the WA Health Network approach: i) development of the Model of Care, ii) policy uptake and, iii) policy implementation (Figure 1). At each stage, stakeholder engagement, through engagement and consultation, is critical. In the first phase, support to develop a Model of Care is provided by officers with the Department of Health. These officers are critical to the development of the policies by providing project management, executive support, policy intelligence, and in some cases content expertise, to ensure the Model of Care meets the needs of the Department and can be used as an effective platform for the implementation of recommendations. Notably, these government officers are considered imperative to the Model of Care development process [40]. A multidisciplinary working group of interested individuals from the Network and the government officer are responsible for ensuring the recommendations of the Model are evidence-informed, by conducting literature reviews, referring to evidence from systematic reviews and engaging with academics/researchers who are cognizant with current evidence. The working group also ensures that the recommendations are appropriate to the WA context; that is, the state’s health system and unique geography. To ensure policies and recommendations remain consumer-centred, each Model of Care is informed by consumer and/or carer input at every stage.

In the second phase, to ensure policy acceptance or ‘buy-in’, from stakeholders, each Model of Care undergoes a comprehensive consultation phase among all Health Network members (individuals and organisations), as well as recognised content leaders and consumers before a final document is released. Comprehensive consultation ensures polices are written and informed *by* state-wide stakeholders *for* state-wide stakeholders. The working group members and the government officer have an important role in engaging with stakeholders to support consultation initiatives and promoting the Model of Care. A range of consultation strategies are employed including meetings, forums, targeted invitations and the use of web-based platforms to provide feedback.

In the third phase, implementation is facilitated by an iterative approach to understanding barriers and enablers to translating recommendations into practice. Effective implementation may also be achieved by integrating various strategies/projects and aligning activity with other contemporary policies or strategic directions, for example, activity-based funding models.

A range of factors are required to achieve desired outcomes in each of these three phases, including.

•Establishment of a group (Network) of multidisciplinary people/organisations who share a common interest in a clinical or health service area, including consumers and carers.

•Organisational support to coordinate the group as well as in kind support from employers of Network members, allowing them time to contribute to Network activities.

•Support from, and formal integration with, Government.

•Development and acceptance of contextually-appropriate policy, creating a platform from which implementation activities can be initiated.

•Seed funding to initiate pilot projects which can subsequently be embedded into sustainable service models.

### Application of a Health Network approach model for addressing service delivery in musculoskeletal health WA

In Australia, musculoskeletal health is recognised as a National Health Priority Area, attributed to the profound social and economic burden of conditions such as osteoarthritis, rheumatoid arthritis, osteoporosis and pain of musculoskeletal origin [41,42]. In recognition of the social burden of musculoskeletal health conditions, a suite of evidence-based and consumer-centred musculoskeletal Models of Care has been developed by the WA Musculoskeletal Health Network. These Models of Care serve as a platform to improve service delivery in musculoskeletal health, specifically for inflammatory arthritis, spinal pain, elective joint replacement surgery for osteoarthritis, and osteoporosis [43-46], and complement a National Service Improvement Framework [47] as well as Models of care from other states, particularly New South Wales (NSW) [48-50]. Consistent with broader policies for chronic diseases [6,51], the WA Models of Care for musculoskeletal health provide recommendations concerning the delivery community-based, interdisciplinary care for consumers and carers across the care continuum. For example, the establishment of community-based, multidisciplinary rheumatology/musculoskeletal health services was identified as a key recommendation in the Inflammatory Arthritis Model of Care [43]. This recommendation highlights a substantial change in historic service delivery in the public health system for this clinical area. In WA, public rheumatology care has been delivered primarily in the tertiary hospitals for residents of the metropolitan area. Establishment of community-based services promotes the transition of care from tertiary hospitals to community facilities to improve ease and equity of access to care, improve cost effectiveness, and ease pressure on tertiary hospitals. Here, we present the implementation of this initiative as a case study to demonstrate the application of a Health Network approach to achieving translation of evidence into policy and practice.

## Case presentation

### Establishment, structure and membership of the WA musculoskeletal Health Network

The WA Musculoskeletal Health Network ( http://www.healthnetworks.health.wa.gov.au/network/musculoskeletal.cfm), one of 18 WA Health Networks, was established in 2006. The strategic direction of the Network is guided, through consultation, by a multidisciplinary (including consumers and carers) Executive Advisory Group (EAG) of 17 members. Activities undertaken by the Network (e.g. developing Models of Care or other policies, undertaking specific projects, developing business cases or policy implementation plans) are performed by multidisciplinary working groups which are convened from the broader Network of members and are led by a clinical ‘champion’ or clinical lead. These working groups report to the EAG. The EAG and working groups are supported by a dedicated Network officer/manager from the Department of Health, within the Division of System Policy and Planning. The Networks do not have access to recurrent funding. At September 2012, there were 3075 stakeholders registered with the WA Health Networks, of which 2713 (88.2%) receive regular communication about Network-related activities. 546 stakeholders are registered with the Musculoskeletal Health Network and 503 (92.1%) receive regular communication about Network activities (Table 1). Models of Care have been developed by the Musculoskeletal Network since 2006, with the most recent Model of Care published in 2011. The Network has also contributed to the development of state-wide strategic frameworks addressing chronic diseases [6,51].

**Table 1:** Multidisciplinary stakeholders represented in the WA Musculoskeletal Health Network ( http://www.healthnetworks.health.wa.gov.au/network/musculoskeletal.cfm)

**Category of stakeholder**	**Examples**
Allied health professionals	Physiotherapists, chiropractors, osteopaths, occupational therapists, psychologists
Carers	Individuals who care for people with chronic health and musculoskeletal conditions
Consumers	Individuals with chronic health conditions and chronic musculoskeletal conditions
Corporate	Health insurance companies (profit and not-for-profit)
Cross-jurisdictional representation	NSW Musculoskeletal Network members
General practitioners	
Inter-departmental representation	WorkCover WA, WorkSafe WA, Disability Services Commission
Medical specialists	Rheumatologists, endocrinologists, orthopaedic surgeons, pain specialists, anaesthetists
Medicare Locals	
Non-government organisations	Arthritis and Osteoporosis WA, Injury Control Council of WA, Council of the Ageing
Nursing	Nurses and nurse practitioners
Pharmacists	Community and hospital pharmacists
Policy makers and planners	Officers across the divisions of the Department, officers from the Area Health Services
Professional Organisations	Australian Physiotherapy Association, Royal Australian College of General Practitioners, Australian Chiropractic Association, Australian Rheumatology Association
Research, Education and Training Organisations	Universities, Research Institutes

### Development of the case study

The authors undertook a review of Network-related activities over the last three years which aligned to the Network objective of delivering evidence-informed health services to consumers with musculoskeletal health conditions. The review focused on outcomes and the processes used to achieve this objective in the context of: i) development of policy; ii) procurement of funding; iii) stakeholder engagement, specifically the identification of partnerships with individuals or organisations which have been associated with policy or project outcomes; iv) publications; and v) projects undertaken by the Network which aligned to implementation of Models of Care. The review was informed by operational reporting undertaken by the Network officer, discussions with leaders of Network working groups, reports from working groups, reports from the EAG, and information gathered from a large stakeholder forum held in 2011 [52].

The review identified a range of activities undertaken by the Network which aligned to development and implementation of the Models of Care for musculoskeletal health, particularly in the areas of establishing services, workforce development and encouraging active self-management by consumers.

### Development and implementation of Models of Care

In WA, four Models of Care have been produced for musculoskeletal health [43-46], all of which are now at the stage of implementation and are supported by broader policy frameworks [6,51,53]. Broader policy frameworks provide the added advantage of raising the profile of Models of Care within government and better integrating them with the broader health policy directions. Implementation projects are being led by local clinical leaders in the areas of spinal pain, inflammatory arthritis, osteoporosis and elective joint replacement surgery for osteoarthritis. The projects are supported by the broader Network and the government officers. We present a specific case related to the implementation of the Inflammatory Arthritis Model of Care [43].

### Implementation the Inflammatory Arthritis Model of Care: community-based, multidisciplinary rheumatology services

Over a three-year period since the completion of the Inflammatory Arthritis Model of Care, strategies to address specific policy implementation challenges have been used to drive implementation of this Model of Care (Table 2), and others. The outcomes of applying the Health Network approach to implementation of this policy are summarised below.

•Development of a consumer-centred and evidence-informed Model of Care [43].

•Identification of essential disease-specific knowledge and clinical skills required by community-based physiotherapists to support a rheumatology service [54,55].

•Development of learning modules for physiotherapists in clinical service delivery (currently in progress) and for all health professionals in delivery of self-management programs. The learning modules will complement a suite of resources being created in Australia to develop workforce capacity in musculoskeletal health, including foundation curricula in rheumatology, clinical skills in ankylosing spondylitis, a post graduate certificate for nurses in musculoskeletal health, and e-resources for the effective self-management of musculoskeletal pain.

•Procurement of funds to support the establishment of a community-based rheumatology service.

•Establishment of partnerships with a local hospital, community-based organisations, universities and private service providers (rheumatologists and general practitioners) to support the community-based service.

•Delivery of self-management and exercise programs for consumers at the implementation site by community-based organisations.

**Table 2:** Challenges and strategies to drive implementation of evidence-informed policy within a government-supported Network model

**Strategy**	**Explanation**	**Working example: establishment of a community-based, multidisciplinary rheumatology service**
*Challenge: taking ownership and responsibility to lead an initiative*		
Identify and support a local clinical leader to drive implementation priorities.	A local clinical leader in a specific area can be identified and encouraged to lead a piece of work which is both of interest to them and aligns with a policy recommendation. Highlighting implementation opportunities to leaders may also facilitate this process. The government officer has a key role in supporting the leader with policy intelligence, linking them with Departmental processes and personnel, and providing project support and stakeholder engagement opportunities.	A local rheumatologist was identified as a clinical leader in promoting the establishment of a community-based rheumatology service in an outer metropolitan area.
*Challenge: knowing where and how to start with a service reform project*		
Map opportunities, barriers and enablers to implementation.	The clinical leader, government officer and other stakeholders identify system barriers and enablers to implementing Model of Care recommendation(s). By exploring the barriers and enablers, a specific project(s) may be developed.	Barriers and enablers were mapped, including:
		Barriers: local workforce limitations (skills, knowledge, volume), establishing new clinical and clerical positions within an area health service, funding limitations especially for consultant salaries, lack of local community-based support services, lack of data to substantiate need, lack of programme evaluation expertise.
		Enablers: opportunity to upskill local clinicians, engage the private sector in clinical service delivery, apply for grants to procure seed funding, partner with non-government providers in community-based service delivery, partner with universities for evaluation expertise.
Develop an evidence-informed and data-driven business case/project plan with longer term implementation/integration strategies identified.	The project leader, working group and government officer develop a data-driven business/project plan to demonstrate need, explicitly identify how the work aligns with policy, and the predicted risk and benefits to the local health system. Partnerships with researchers and intra-departmental agencies (e.g. Epidemiology) are important for developing a robust and mutually agreeable business case/project plan. Providing a description of how the initiative links with the broader policy directions and funding models is critical to increasing the likelihood of acceptance from decision makers and opportunities to maintain sustainability.	Project plan developed, supported by data concerning the number of referrals to tertiary hospital-based rheumatology departments according to geographic areas in the state. Geographic areas of need could then easily be identified. The plan included strategies to upskill local clinicians, engage the private sector to provide clinical services (allied health and rheumatologists), and partner with other community-based organisation to provide local services (e.g. self-management and exercise programs) in the area identified as the site for implementation.
*Challenge: attracting buy-in from other stakeholders for gaining assistance and influencing decision makers*		
Establish a local working group of interested people from the Network.	Individuals who express an interest in the project(s) are invited to join a working group, share ideas and lead sub-components of the project in a distributed leadership model. The government officer and an emerging group of stakeholders identify others to contribute to the project.	Invite individuals with interest and skill sets applicable to establishing a service (clinicians, consumers, policy makers, business managers, researchers). The importance of the end goal and how the proposed iterative processes aligned with individuals’ interests were emphasised.
Maintain government officer support.	The government officer provides project management support and executive support to ensure that project activities align with policy, provide in kind resources and identify opportunities for integrating with other projects and relevant stakeholders.	Utilise knowledge of government officer to link project objectives with other opportunities within government (e.g. funding programs for community-based care) and attract buy-in from other stakeholders.
Seek executive support.	Executive support to progress the plan is sought. This may involve approval to seek funding, identify partnership opportunities and promote the project concept to other executives. Executive support also facilitates engagement with, and support from, middle management.	Project plans presented to executives for support. Regular briefings were provided on progress of implementation.
Engage support from broader stakeholder group (the Community of Practice).	Maximising buy-in from other stakeholders (e.g. the clinical community, area health services, policy-makers) on a particular project can be facilitated by promoting a project objective and working group to these individuals. This may achieved through Network forums or e-newsletters. A broad support base help to forge and maintain partnerships and sustain the political will to support the initiative.	Once executive support received, the project was promoted through the Network via meetings, e-newsletters and a major stakeholder forum [52].
Identify cross-sectoral partnerships in supporting implementation strategies.	Engage with organisations, such as universities and non-government organisations, to promote the project concept and canvass interest in partnership opportunities. Non-government organisations are critical partners for community-based and consumer-centred projects due to their various community-based programs and established relationships with both professional bodies and consumers.	Project team expanded to include partnerships with other organisations including universities for research and evaluation expertise, WA Community Physiotherapy Services for allied health services ( http://www.health.wa.gov.au/cps/about/), Arthritis and Osteoporosis WA for consumer services (e.g. provision of self-management programs and consumer engagement) ( http://www.arthritiswa.org.au/), and private service providers for clinical rheumatology services.
*Challenge: gaining preliminary financial support*		
Procure funding or in-kind support for initial establishment.	Submit funding applications for competitive grants, government grants and pooled funding initiatives between partner organisations (e.g. government, non-government organisations, universities).	Grant applications submitted for funding specific components of the implementation project. A grant was awarded for a programme designed to upskill clinicians while government funding was awarded to establish a service and appoint a project manager.
Ensure service agreements with non-government organisations reflect contemporary policy.	Support for implementation activities may also be provided by non-government organisations (e.g. delivery of community-based self-management programmes). Aligning service agreements between government and such organisations supports the implementation of policy recommendations and projects.	A service agreement between the Department of Health and a non-government provider ensures that local community-based services are available to consumers at the implementation site and other potential replication sites.
*Challenge: securing sustainability*		
Pilot and evaluate a model.	Undertake the proposed project (e.g. a service model) and ensure evaluation is underpinned by sound research principles. Partnerships with research organisations are important to for achieving robust evaluation methods.	An evaluation framework has been established to monitor consumer-centred and system-centred outcomes.
Disseminate results, e.g. through publications, reports, presentations.	Communicate the outcomes of the evaluation and opportunities and barriers for achieving programme sustainability.	
Integrate parallel projects to build cumulative change momentum in an area and avoid duplication of efforts.	Where possible, link specific policy implementation projects through communication and project promotion. This may be achieved through forums, e-bulletins and government officers having knowledge of discrete pieces of work being developed and undertaken.	Linked projects with evaluations include the establishment of a rheumatology service at a specific site, clinical workforce upskilling in rheumatoid arthritis (state and national projects) and development of self-management programs for consumers and health professionals in disease-specific musculoskeletal health conditions.

### Integration across Network projects

Across the four Models of Care, many of the Network projects were integrated to achieve the broad aim of improved access to, and delivery of, services for consumers with musculoskeletal health conditions. The strategy to integrate Network-led projects enables an incremental and coordinated approach to more successfully addressing challenges around health system redesign. This exemplifies the value of moving from silos of independent activity in clinical, policy and research arenas, to a co-ordinated approach facilitated through a Network model. Examples of other Network-led projects linked with recommendations in the Models of Care and which are purposely linked to the community-based rheumatology service are summarised below.

•Implementing a system inversion model in a tertiary hospital pain medicine unit, such that consumers with persistent pain attend pre-clinic group education sessions prior to an assessment by an interdisciplinary pain medicine team [56]. This model has demonstrated cost savings for the health service, reduced waiting lists, and increased active management by consumers for their pain conditions. The model is now recurrently funded and being rolled out into primary care settings.

•Providing interdisciplinary professional development in musculoskeletal pain to health care professionals in urban and rural WA, resulting in a sustained improvement in adoption of evidence-based practices for clinical management [57].

•Providing evidence-based self-management training and skills to consumers with musculoskeletal pain in rural WA, resulting in improved beliefs about low back pain and highlighting the need for sustained reinforcement strategies of positive self-management skills in rural WA [58].

•Undertaking an audit of beliefs and likely clinical practice behaviours in the context of low back pain among final year allied health and medical students in WA in an effort to assess local workforce readiness to deliver health services and information in a guideline-consistent manner [59]. Findings will be used to influence university curriculum for medical and allied health students to ensure optimal alignment with evidence.

•Trialling a novel model of post-operative follow-up after joint replacement surgery to minimise costs to health services while maintaining quality and patient satisfaction (in progress) and determining hospital discharge information needs among general practitioners [60].

•Development and evaluation of written and e-resources for consumers with musculoskeletal pain in WA, for example in back pain [61] (in progress).

•Development of a state-wide access algorithm for osteoporosis therapies (in progress).

## Discussion

### Disease and system-level outcomes achieved from adopting a Health Network approach to evidence-informed policy development and implementation

While the implementation stage of the WA Models of Care varies, overall, the use of a Network approach has contributed to the success of developing and implementing recommendations from these policies. The Network approach we describe for developing evidence-informed policies and their translation into practice has achieved some important outcomes for WA. For example, in addition to achieving specific health system improvements in musculoskeletal health, most notably for inflammatory arthritis [54,55] and spinal pain [56-59,62], and influencing national health reform debates and strategies in these areas [42,63], the WA Models of Care are now recognised as critical components to health-related policy, planning and funding decisions in WA. Specifically, Models of Care are used by non-government organisations to set strategic directions and inform development of business cases, by state health services to improve existing clinical service models or establish new models, by state infrastructure and clinical services planning divisions to reach decisions on the locations and facilities required to deliver services [64], and in state funding allocation decisions. Similar outcomes are being realised in NSW with the development and release of Models of Care for musculoskeletal health in that state through the NSW Musculoskeletal Network [48-50].

In the context of musculoskeletal health, important outcomes have been achieved in WA which are likely to promote sustainability of the WA Health Network approach and foster an ongoing policy-into-practice research agenda. The sustainability of the Network model itself is most likely related to the achievement of positive outcomes (organizational success), for example the establishment of a community-based rheumatology service and reconfiguration of service delivery in pain medicine units [56]; social inclusion amongst a community of stakeholders (social inclusion) [65]; and emerging evidence for improved consumer health [56,58], all of which build resilience and foster sustainability for the Network [66]. These outcomes underline the potential system improvements and consumer health gains achievable using a Health Network model with a phased and coordinated implementation approach that involves cross-discipline stakeholders and consumers, and highlight the importance of developing evidence-informed and contextually appropriate policy to enable contextually feasible implementation initiatives [38]. Strategies amenable to Networks may therefore prove successful in overcoming previous attempts by government to tackle specific clinical issues and that have historically been less effective and sustainable.

### Why apply a Network approach to service delivery for chronic diseases?

Implementing health service delivery changes to better address chronic health conditions is challenging due to the complex aetiology of these conditions, the multiple stakeholders required in order to achieve optimal consumer outcomes, and the historic design of healthcare systems to address acute healthcare needs in hospitals rather than long-term healthcare needs in community settings. From an implementation perspective, the Health Network approach is effective because it i) connects stakeholders from across the health sector – researchers, clinicians, consumers, policy-makers and so on, and ii) provides mechanisms and pathways for action. Without the Health Network model, formalised and regular connections between these stakeholders would not otherwise occur, as highlighted recently [15]. In the context of musculoskeletal health, this approach is particularly useful since the majority of service delivery in this clinical area occurs in ambulatory care settings; hence, effective integration among stakeholders across primary care and hospital-based care is critical to achieving seamless service delivery for consumers. The case study described in this paper involved multiple stakeholders, in particular the establishment of a partnership model among researchers, clinicians and policymakers. The effectiveness of the WA Health Network approach is not only attributable to cumulative implementation efforts, that is by integrating several projects, but also reflects successful engagement with local, domain-specific leaders and support from the broader Network. The identification of local, domain-specific leaders, often termed ‘champions’, supported within a distributed leadership framework, is well recognised as effective model to promote change, motivate participation from others and drive complex and multidimensional projects [32,67,68] and has also been identified as a facilitator to successful evidence integration and translation in health care policy [13]. This type of organisational model, particularly the support of local leaders, has been shown to be effective across different health areas [69,70].

### Using Health Networks to embed research into policymaking

The WA Health Network approaches align strongly with the recognised need for research evidence to inform and optimise public health policymaking [71,72], especially in the context of chronic health conditions [6,51]. In Australia, the National Health and Medical Research Council (the nation’s main funding source for health research) also recognises this need and now supports partnership projects among research leaders and policy leaders [73], while at a state level, grants are offered to enable the translation of policy into practice through the State Health Research Advisory Council’s Research Translation scheme [74]. While opportunities to explore evidence translation into policy and policy translation into practice are emerging in a research context, and may be supported by a Health Network approach, resources to support broader implementation of evidence-based programs and policy recommendations are less attainable.

### Future directions and limitations

As evidence for the strength of Network (or partnership)-based health improvement initiatives accumulates, access to funding opportunities and organisational support, particularly that of middle-management, to drive the implementation of service reforms will be important because effective program implementation is difficult and often requires specific expertise combined with organisational support to achieve sustainable success. For example, the effectiveness of various program implementation initiatives will vary; such that a well-supported, average program may well outperform a poorly supported, high-quality program [75]. Therefore, developing implementation expertise within the Health Networks will be important for delivering innovative and sustainable health reforms in WA, particularly in rural WA [76]. Further the Health Networks will need to constantly adapt and respond to the emerging Australian health reforms and develop innovative means to maintain effective communication between stakeholders, for example through electronic modes of communication, such as social media, as stakeholders’ expectations for modes of communicating change from traditional face-to-face and email exchanges. A further challenge for the Health Networks will be to maintain alignment of the Models of Care with contemporary evidence. A notable limitation of the Network process is the time taken to develop a Model of Care or implementation plan, usually in the order of 1–2 years. While effective consultation is time-consuming, protracted development processes may stymie ability to respond quickly to emerging opportunities and disenfranchise members due to the perception of inaction and unnecessary processes.

## Conclusion

Health reform is not easy, and as such there is no simple solution for implementing change with the aim of improving health outcomes and meeting sustainability demands. Using the currency of social interaction through engaging Health Networks is one framework that appears to offer potential and requires further investigation. While much of the research into the effectiveness of Networks and communities of practice has focussed on their establishment and scope of work [19], and some more contemporary findings on health system safety and quality [20], evaluation of Network-related effectiveness for improving patient outcomes should now be explored. In WA, the Health Networks have provided a sustainable mechanism to meaningfully engage consumers, carers, clinicians and other stakeholders; provided a forum to exchange ideas, information and evidence; and collaboratively plan and deliver evidence-based and contextually-appropriate health system improvements for consumers.

## Abbreviations

CoP: Community of practice; MoC: Model of care, NSW, New South Wales; WA: Western Australia.

## Competing interests

The authors declare no conflicts of interest. AMB works as a senior government officer, responsible for coordinating and overseeing of the WA Musculoskeletal Health Network, and other Networks. SCBT was former Chief Medical Officer for the state of Western Australia and as the executive of the Health Networks was responsible for their initiation and strategic direction. HS and MC have been involved in the development and implementation of the Models of Care.

## Authors’ contributions

AMB, PB and HS developed the conceptual framework for the manuscript (focus, scope, content) and prepared the initial drafts, principally led by AMB. MC and SCBT further developed the initial drafts. All authors reviewed and approved the final version of the manuscript.

## Pre-publication history

The pre-publication history for this paper can be accessed here:

http://www.biomedcentral.com/1472-6963/12/394/prepub

## References

[B1] Australian Institute of Health & Welfare Australia's Health 2010 2010 Canberra: AIHW

[B2] Thrall JH Prevalence and costs of chronic disease in a health care system structured for treatment of acute illness Radiology 2005 235 9 12 10.1148/radiol.2351041768 15798162

[B3] World Health Organization Global Status Report on Non-Communicable Diseases 2010 2011 Geneva: WHO

[B4] Yach D Hawkes C Gould CL Hofman KJ The global burden of chronic diseases: overcoming impediments to prevention and control JAMA 2004 291 2616 2622 10.1001/jama.291.21.2616 15173153

[B5] National Health Priority Action Council National Chronic Disease Strategy 2005 Canberra: Australian Government Department of Health and Ageing

[B6] Department of Health (Western Australia) WA Chronic Health Conditions Framework 2011–2016 2011 Perth: Health Networks Branch

[B7] Lavis JN Posada FB Haines A Osei E Use of research to inform public policymaking Lancet 2004 364 1615 1621 10.1016/S0140-6736(04)17317-0 15519634

[B8] Buchbinder R Osborne RH Ebeling PR Wark JD Mitchell P Wriedt C Graves S Staples MP Murphy B A randomized trial of vertebroplasty for painful osteoporotic vertebral fractures New Engl J Med 2009 361 557 568 10.1056/NEJMoa0900429 19657121

[B9] Kallmes DF Comstock BA Heagerty PJ Turner JA Wilson DJ Diamond TH Edwards R Gray LA Stout L Owen S A randomized trial of vertebroplasty for osteoporotic spinal fractures N New Engl J Med 2009 361 569 579 10.1056/NEJMoa0900563 PMC2930487 19657122

[B10] Staples MP Kallmes DF Comstock BA Jarvik JG Osborne RH Heagerty PJ Buchbinder R Effectiveness of vertebroplasty using individual patient data from two randomised placebo controlled trials: meta-analysis BMJ 2011 343 d3952 10.1136/bmj.d3952 21750078 PMC3133975

[B11] Cochrane Effective Practice and Organisation of Care (EPOC) Group EPOC Reviewshttp://epoc.cochrane.org/epoc-reviews

[B12] McMaster Health Systems Evidence databasehttp://www.mcmasterhealthforum.org/healthsystemsevidence-en

[B13] Mitton C Adair CE McKenzie E Patten SB Waye Perry B Knowledge transfer and exchange: review and synthesis of the literature Milbank Q 2007 85 729 768 10.1111/j.1468-0009.2007.00506.x 18070335 PMC2690353

[B14] Lavis JN Oxman AD Moynihan R Paulsen EJ Evidence-informed health policy 1 - synthesis of findings from a multi-method study of organizations that support the use of research evidence Implement Sci 2008 3 53 10.1186/1748-5908-3-53 19091107 PMC2621242

[B15] Haynes AS Derrick GE Redman S Hall WD Gillespie JA Chapman S Sturk H Identifying trustworthy experts: how do policymakers find and assess public health researchers worth consulting or collaborating with? PLoS One 2012 7 e32665 10.1371/journal.pone.0032665 22403693 PMC3293848

[B16] Li LC Bombardier C Setting priorities in arthritis care: Care III Conference J Rheumatol 2006 33 1891 1894 16960949

[B17] Nielsen CP Lauritsen SW Kristensen FB Bistrup ML Cecchetti A Turk E European Network Hlth Technol A Involving stakeholders and developing a policy for stakeholder involvement in the European network for Health Technology Assessment, EUnetHTA Int J Technolog Assess Health Care 2009 25 84 91 10.1017/S0266462309990729 20030895

[B18] Ranmuthugala G Plumb JJ Cunningham FC Georgiou A Westbrook JI Braithwaite J How and why are communities of practice established in the healthcare sector? A systematic review of the literature BMC Health Serv Res 2011 11 10.1186/1472-6963-11-273 PMC3219728 21999305

[B19] Ranmuthugala G Cunningham FC Plumb JJ Long J Georgiou A Westbrook JI Braithwaite J A realist evaluation of the role of communities of practice in changing healthcare practice Implement Sci 2011 6 49 10.1186/1748-5908-6-49 21600057 PMC3120719

[B20] Cunningham FC Ranmuthugala G Plumb J Georgiou A Westbrook JI Braithwaite J Health professional networks as a vector for improving healthcare quality and safety: a systematic review BMJ Qual Saf 2012 21 239 249 10.1136/bmjqs-2011-000187 PMC3285140 22129933

[B21] Lewis JM Being around and knowing the players: Networks of influence in health policy Social Sci Med 2006 62 2125 2136 10.1016/j.socscimed.2005.10.004 16289737

[B22] Wenger E McDermott R Snyder WM Cultivating Communities of Practice 2002 Boston, MA: Harvard Business School Press

[B23] Bourhis A Dube L 'Structuring spontaneity': investigating the impact of management practices on the success of virtual communities of practice J Inform Sci 2010 36 175 193 10.1177/0165551509357861

[B24] Curran JA Murphy AL Abidi SSR Sinclair D McGrath PJ Bridging the gap: knowledge seeking and sharing in a virtual community of emergency practice Eval Health Professions 2009 32 314 327 10.1177/0163278709338570 19696084

[B25] Henrickson M Policy challenges for the pediatric rheumatology workforce: Part III. the international situation Pediatric Rheumatol 2011 9 10.1186/1546-0096-9-26 PMC3184619 21910871

[B26] Mellins ED Rider LG Clinical research networks: a step towards evidence-based practice in pediatric rheumatology Nature Clin Prac Rheumatol 2007 3 59 59 10.1038/ncprheum0405 17299440

[B27] Giles M Van Der Kallen J Parker V Cooper K Gill K Ross L McNeill S A team approach: implementing a model of care for preventing osteoporosis related fractures Osteoporos Int 2011 22 2321 2328 10.1007/s00198-010-1466-0 21046071

[B28] Liu L Krailo M Reaman GH Bernstein L Childhood cancer patients' access to cooperative group cancer programs: a population-based study Cancer 2003 97 1339 1345 10.1002/cncr.11192 12599243

[B29] Lih A Nandapalan H Kim M Yap C Lee P Ganda K Seibel MJ Targeted intervention reduces refracture rates in patients with incident non-vertebral osteoporotic fractures: a 4-year prospective controlled study Osteoporos Int 2011 22 849 858 10.1007/s00198-010-1477-x 21107534

[B30] Spence K Henderson-Smart D Closing the evidence-practice gap for newborn pain using clinical networks J Paed Child Health 2011 47 92 98 10.1111/j.1440-1754.2010.01895.x 21091580

[B31] Fung-Kee-Fung M Watters J Crossley C Goubanova E Abdulla A Stern H Oliver TK Regional collaborations as a tool for quality improvements in surgery: a systematic review of the literature Ann Surg 2009 249 565 572 10.1097/SLA.0b013e31819ec608 19300234

[B32] Greene A Pagliari C Cunningham S Donnan P Evans J Emslie-Smith A Morris A Guthrie B Do managed clinical networks improve quality of diabetes care? Evidence from a retrospective mixed methods evaluation Qual Safety Health Care 2009 18 456 461 10.1136/qshc.2007.023119 19955457

[B33] Totlandsdal AI Fudge N Sanderson EG van Bree L Brunekreef B Strengthening the science-policy interface: experiences from a European Thematic Network on Air Pollution and Health (AIRNET) Environ Sci Policy 2007 10 260 266 10.1016/j.envsci.2007.01.003

[B34] Clemson L Finch CF Hill KD Lewin G Fall prevention in Australia: policies and activities Clin Geriatric Med 2010 26 733 749 10.1016/j.cger.2010.07.002 20934619

[B35] Health Reform Committee WA A Healthy Future for Western Australians. Report of the Health Reform Committee 2004 Department of Health, Government of Western Australia

[B36] Department of Health Clinical Networks in Western Australia. Background Paper 2005 Perth: Department of Health, Government of Western Australia

[B37] Borzel T Organising Babylon - on the different conceptions of policy networks Public Admin 1998 76 253 273 10.1111/1467-9299.00100

[B38] Aarons GA Sommerfeld DH Walrath-Greene CM Evidence-based practice implementation: the impact of public versus private sector organization type on organizational support, provider attitudes, and adoption of evidence-based practice Implement Sci 2009 4 10.1186/1748-5908-4-83 PMC2813227 20043824

[B39] Department of Health (Western Australia) Model of Care Overview and Guidelines 2007 Perth: Health Networks Branch

[B40] Cunningham FC Ranmuthugala G Westbrook JI Braithwaite J Net benefits: assessing the effectiveness of clinical networks in Australia8th International Organisation Behaviour in Healthcare Conference 2012 Dublin 10.1186/1748-5908-7-108 PMC3541150 23122000

[B41] Australian Institute of Health & Welfare Arthritis and musculoskeletal conditions in Australia 2005 Canberra: AIHW

[B42] Australian and New Zealand College of Anaesthetists National Pain Strategy 2010 MelbourneFaculty of Pain Medicine

[B43] Department of Health (Western Australia) Inflammatory Arthritis Model of Care 2009 Perth: Health Networks

[B44] Department of Health Western Australia Spinal Pain Model of Care 2009 Perth: Health Networks Branch, Department of Health, Western Australia;

[B45] Department of Health (Western Australia) Osteoporosis Model of Care 2011 Perth: Health Networks Branch

[B46] Department of Health (Western Australia) Elective Joint Replacement Service Model of Care 2010 Perth: Health Networks Branch

[B47] National Health Priority Action Council National Service Improvement Framework for Osteoarthritis, Rheumatoid Arthritis and Osteoporosis 2006 Canberra: Australian Government Department of Health and Ageing

[B48] NSW Agency for Clinical Innovation Musculoskeletal Network: NSW Model of Care for Osteoporotic Refracture Prevention 2011 Sydney: NSW Agency for Clinical Innovation

[B49] NSW Agency for Clinical Innovation Musculoskeletal Network: Model of Care for Paediatric Rheumatology (draft) 2012 Sydney: NSW Agency for Clinical Innovation

[B50] NSW Agency for Clinical Innovation Musculoskeletal Network: Osteoarthritis Chronic Care Program Model of Care 2012 Sydney: NSW Agency for Clinical Innovation

[B51] Department of Health (Western Australia) WA Chronic Conditions Self-Management Strategic Framework 2011–2015 2011 Perth: Health Networks Branch

[B52] Department of Health (Western Australia) Musculoskeletal Health Network Stakeholder's Forum Report, 13 September 2011 2011 Perth: Health Networks Branch

[B53] Department of Health (Western Australia) WA Primary Health Care Strategy 2011 Perth: Health Networks Branch

[B54] Briggs AM Fary RE Slater H Bragge P Chua J Keen HI Chan M Disease-specific knowledge and clinical skills required by community-based physiotherapists to co-manage patients with rheumatoid arthritis Arthritis Care Res 2012 64 1514 1526 10.1002/acr.21727 22556156

[B55] Fary RE Slater H Chua J Briggs A Translating policy into practice for community-based management of rheumatoid arthritis: Targeting professional development needs among physiotherapists Int J Rheumatol 10.1155/2012/240689 PMC3502858 23209474

[B56] Davies S Quintner J Parsons R Parkitny L Knight P Forrester E Roberts M Graham C Visser E Antill T Preclinic group education sessions reduce waiting times and costs at public pain medicine units Pain Med 2011 12 59 71 10.1111/j.1526-4637.2010.01001.x 21087401

[B57] Slater H Davies SJ Parsons R Quintner JL Schug SA A policy-into-practice intervention to increase the uptake of evidence-based management of low back pain in primary care: a prospective cohort study PLoS One 2012 7 e38037 10.1371/journal.pone.0038037 22662264 PMC3360643

[B58] Slater H Briggs AM Bunzli S Davies SJ Smith AJ Quintner J Engaging consumers living in remote areas of Western Australia in the self-management of back pain: a prospective cohort study BMC Musculoskelet Disord 2012 13 69 10.1186/1471-2474-13-69 22578207 PMC3439262

[B59] Briggs AM Slater H Smith AJ Parkin-Smith GF Watkins K Chua J Low back pain-related beliefs and likely practice behaviours among final-year cross-discipline health students Eur J Pain 2012 (epub ahead of print) 10.1002/j.1532-2149.2012.00246.x 23139051

[B60] Briggs AM Lee N Sim M Leys TJ Yates PJ Hospital discharge information after elective total hip or knee joint replacement surgery: A clinical audit of preferences among general practitioners Australasian Med J 2012 5 544 550 10.4066/AMJ.2012.1471 PMC3494827 23173019

[B61] Consumer's guide to managing back pain http://www.healthnetworks.health.wa.gov.au/docs/2010_BackPain.pdf

[B62] Davies SJ Hayes C Quintner JL System plasticity and integrated care: informed consumers guide clinical reorientation and system reorganization Pain Med 2011 12 4 8 10.1111/j.1526-4637.2010.01016.x 21143757

[B63] Cousins MJ Unrelieved pain: a major healthcare priority Med J Aust 2012 196 373 374 10.5694/mja12.10181 22471529

[B64] Department of Health (Western Australia) WA Health Clinical Services Framework 2010–2020 2009 Perth: Health System Improvement Unit

[B65] Braithwaite J Westbrook JI What makes the health system tick? Int J Qual Health Care 2010 22 1 2 10.1093/intqhc/mzp055 19955109 PMC2803010

[B66] Weick K Sutcliffe K Managing the unexpected: resilient performance in an age of uncertainty 2007 San Francisco, CA: John Wiley & Sons, Inc

[B67] Barrett LL Plotnikoff RC Raine K Organizational leadership and its relationship to regional health authority actions to promote health J Health Organ Manag 2007 21 259 282 10.1108/14777260710751735 17713187

[B68] Doumit G Gattellari M Grimshaw J O'Brien MA Local opinion leaders: effects on professional practice and health care outcomes Cochrane Database Syst Rev 2007 CD000125 17253445 10.1002/14651858.CD000125.pub3

[B69] Eskicioglu C Gagliardi A Fenech DS Victor CJ McLeod RS Can a tailored knowledge translation strategy improve short term outcomes? A pilot study to increase compliance with bowel preparation recommendations in general surgery Surgery 2011 150 68 74 10.1016/j.surg.2011.02.010 21596413

[B70] Kitson AL The need for systems change: reflections on knowledge translation and organizational change J Advanced Nursing 2009 65 217 228 10.1111/j.1365-2648.2008.04864.x 19032518

[B71] Ham C Hunter DJ Robinson R Evidence based policymaking BMJ 1995 310 71 72 10.1136/bmj.310.6972.71 7833721 PMC2548491

[B72] Hanney SR Gonzalez-Block MA Buxton MJ Kogan M The utilisation of health research in policy-making: concepts, examples and methods of assessment Health Res Policy Syst 2003 1 2 10.1186/1478-4505-1-2 12646071 PMC151555

[B73] NHMRC Partnership Projects http://www.nhmrc.gov.au/grants/apply-funding/partnerships-better-health/partnerships-projects

[B74] Research Development Funding and Research Supporthttp://www.health.wa.gov.au/researchdevelopment/funding/index.cfm

[B75] Durlak JA DuPre EP Implementation matters: a review of research on the influence of implementation on program outcomes and the factors affecting implementation Amer J Community Psychol 2008 41 327 350 10.1007/s10464-008-9165-0 18322790

[B76] Briggs AM Slater H Bunzli S Jordan JE Davies SJ Smith AJ Quintner JL Consumers’ experiences of back pain in rural Western Australia: access to information and services, and self-management behaviours BMC Health Serv Res 2012 12 357 10.1186/1472-6963-12-357 23057669 PMC3494578

